# Proceedings of Veterinary Societies

**Published:** 1889-10

**Authors:** 


### PROCEEDINGS OF VETERINARY SOCIETIES.

---

United States Veterinary Medical Association.—The Twenty-sixth Annual Meeting was held at the Wilson Building, Brooklyn, New York, on the 17th of September, 1889.

President Huidekoper convened the session at 11 A. M., and on roll-call the following members responded to their names: Drs. Barrow, Berns, Geo. Bridges, Burden, Clement, Coates, Cuff, Dixon, Wm. Dougherty, Faust, Goentner, Gill, Hopkins, Hoskins, Howard, Huidekoper, Lyman, Lowe, Michener, Miller, L. McLean, R. A. McLean, Middleton, Mustoe, McKee, Newman, Pendry, Jas. L. Robertson, A. K. Robertson, Wm. Rose, Thos. B. Rayner, Stickney, Winchester, Wray, Weber.

The recommendations of the Comitia Minora were on motion approved, including the election of the following list of new members:—

Dr. E. A. Parsons, New York City; Dr. W. T. Russell, Nashua, N. H.; Dr. H. P. Eves, Wilmington, Del.; Dr. G. A. Lathrop, Binghamton, N.Y.; Dr. A. L. Hummel, Philadelphia, Pa.; Dr. Daniel Lemay, Fort Leavenworth, Kansas; Dr. G. R. Hartman, Philadelphia, Pa.; Dr. T. L. Russell, Orono, Maine; Dr. A. G. Hill, East Boston, Mass.; Dr. H. B. Adair, Kansas City, Mo.; Dr. W. H. Bronnell, New Bedford, Mass.; Dr. W. H. Hitchings, East Somerville, Mass.; Dr. S. Atchison, Brooklyn, N. Y.; Dr. R. S. Stanwood, Freehold, N. J.; Dr. Robert Richards, New York City; Dr. Frank Winant, Greenfield, N. Y.; Dr. F. C. Herbert, Marlboro, N. J.; Dr. Wm. A. Birch, Philadelphia, Pa.; Dr. Thomas E. Maloney, Central Falls, R. I.; Dr. Wm. T. Walsh, New York City; Dr. R. T. Churchill, North Bergen, N. J.; Dr. G. Grimshaw, Kingston, Canada; Dr. Wm. Wappell, Pleasant Valley, Pa.; Dr. Charles J. Weidner, Hilltown, Pa.; Dr. Samuel W. Mathews, Wawa, Pa.; Dr. Porte Crego, Sugar Grove, Ill.; Dr. G. G. Pearson, Philadelphia, Pa.; Dr. Henry Vanderhorst, Mount Vernon, N. Y.; Dr. W. H. Kelly, Albany, N. Y.; Dr. W. H. Robinson, New York City; Dr. Chas. A. Mackey, New York City; Dr. I. F. Page, Manchester, Vermont; Dr. E. M. Osborne, East Hampton, L. I.; Dr. Charles H. Perry, Brooklyn, N. Y.; Dr. James S. Gilbert, Portland, Ind.; Dr. Geo. H. Roberts, East Shelby, N. Y.; Dr. S. S. Moyer, Hilltown, Pa.; Dr. W. B. Prothero, Horton, Pa.; Dr. August Jasme, Atlanta, Ga.; Dr. B. G. Orlopp, Indianapolis, Ind.

The names of Dr. T. W. Spranklin, Baltimore, Md., and Robert C. Jones, Port Jefferson, L. I., were ordered to be stricken from the rolls, for violation of the code of ethics.

The Treasurer's report, showing a balance on hand of $694.18 was ordered spread upon the books.

The Secretary reported the collection of $478.00 in dues and initiation fees, and an expenditure of $426.75 during the past year.

Under the head of Reports of Committees, Dr. W. J. Coates, chairman of Committee on Intelligence and Education, made a very suggestive commentation on the various movements throughout the veterinary world, and what should be the duty and work of this Association. The report was received and referred to the Publication Committee.

Dr. Clements, chairman of the Committee on Contagious and Infectious Diseases, made a voluminous report on the extent of these diseases in the United States, touching upon the whole category in a very suggestive manner. He urged the dropping of the term "Contagious" as applicable to a certain line of diseases, and the substitution of the word "Miasmatic."

The committee on Revision of the Constitution and By-Laws, offered the result of their work, making a complete remodeling of the old plan, and after a few alterations it was adopted and ordered printed.

There were fifteen applicants for membership, and the names of Prof. J. H. Raymond, Brooklin, N. Y., and Prof. H. M. Biggs, New York City, offered for honorary membership.

At 1 P. M., the Association adjourned for one hour to partake of a lunch generously prepared for the Association by the Long Island Veterinary Medical Association.

The election of officers for the ensuing year resulted in the following choice: President, Dr. C. B. Michener, New York City; Vice President, Dr. A. W. Clements, Baltimore, Md.; Secretary, Dr. W. Horace Hoskins, 12 S. 37th St., Philadelphia, Pa.; Treasurer, Dr. Jas. L. Robertson, New York City.

The newly elected officers were then escorted to their chairs, and on retiring from the chair of President, Prof. Huidekoper gave one of the most thorough reviews of the twenty-five years' work of the Association that has ever been prepared. He did not believe that the work of representing the profession in the United States had been as fully performed as the responsibility demanded; neither did he think that the field had been properly covered, or the national questions of importance to the veterinary world so handled as to make the Association a force and power in the country. His resumé bristled with just criticisms, and its study and consideration by each member should be weighed, and better fruits will be brought forth in the next quarter of a century.

Dr. Michener then accepted the honor placed upon him, in fitting words, pledging his untiring efforts in promoting the welfare of the Association, and the betterment of the profession.

The President appointed Drs. Winchester, Howard and Stickney a committee to draft suitable resolutions on the deaths of our late members, Dr. E. F. Thayer, Newton, Mass., and Dr. Chas. L. Moulton, Washington, D.C.

Dr. C. P. Lyman offered a resolution for the establishment of a central, legalized body of veterinarians in this Association. The matter was referred to a special committee consisting of members Lyman, Huidekoper and Jas. L. Robertson, to report at the next meeting.

Dr. Faust had an ably prepared paper on "Veterinary History of the Different Ages," but the lateness of the hour compelled its incomplete reading, and by motion it was referred to the Publication Committee.

The future meetings of the Association will be but once a year, and will be for two or more days in September, which new feature promises a wider range of better work for the profession.

The meeting adjourned at 7 P. M., to partake of one of the most sumptuous banquets of good things, enriched and enlivened by a list of toasts, responded to by Prof. Raymond, Drs. R. S. Huidekoper, Stickney, Hoskins, Michener, Berns, Hon. Henry A. Moore, and others, well and wisely directed by the popular and genial toast master, Dr. W. B. E. Miller, after which to the strains of pleasant music the banqueters separated, voting it one of the most important and successful meetings in the history of the Association, and highly complimentary of the untiring zeal and energy of the local committee of arrangements of the Long Island Veterinary Medical Association.

W. HORACE HOSKINS, *Secretary*.

